# The identity of *Albuca caudata* Jacq. (Hyacinthaceae) and a description of a new related species: *A. bakeri*

**DOI:** 10.3897/phytokeys.5.1166

**Published:** 2011-07-27

**Authors:** Mario Martínez-Azorín, Manuel B. Crespo, Anthony P. Dold, Nigel P. Barker

**Affiliations:** 1 CIBIO (Instituto Universitario de la Biodiversidad), Universidad de Alicante, P.O. Box 99, E-03080 Alicante, Spain; 2 Selmar Schonland Herbarium, Department of Botany, Rhodes University, Grahamstown 6140, South Africa; 3 Department of Botany, Rhodes University, Grahamstown, 6140, South Africa

**Keywords:** *Albuca bakeri* sp. nov., *Albuca caudata*, distribution, taxonomy, typification

## Abstract

The name *Albuca caudata* Jacq. has been widely misunderstood or even ignored since its description in 1791. After studying herbarium specimens and living populations in South Africa, plants fitting Jacquin´s concept of that species are found to be widely distributed in the Eastern Cape, mainly in the Albany centre of Endemism. Furthermore, some divergent specimens matching Baker´s concept of *Albuca caudata* are described as a new related species: *Albuca bakeri*. Data on typification, morphology, ecology, and distribution are reported for both taxa. Affinities and divergences with other close allies are also discussed.

## Introduction

The genus *Albuca* L. is accepted to include about 60 species in recent treatments (cf. Speta 1998; Manning et al. 2002), although up to 131 when considered in a wider sense (Phillips 1951), with over 180 names available in its traditional sense (cf. IPNI 2011). Recent molecular works have however considered the circumscription of *Albuca* in quite different ways. On the one hand, Manning et al. (2009) enlarged the traditional concept of *Albuca* to include other genera such as *Stellarioides* Medik., *Coilonox* Raf., *Trimelopter* Raf. and *Battandiera* Maire, reaching a total of 110–180 species, and hence being very heterogeneous in morphology. On the other, Martínez-Azorín et al. (2011) recovered the traditional concept of *Albuca* on the basis of phylogenetic, morphologic and biogeographic evidences.

Taxa of *Albuca* in its traditional sense (cf. Martínez-Azorín et al. 2011) are distributed mainly in southern and eastern Africa, with only a few species extending to Ethiopia, Saudi Arabia and north of Chad and Nigeria. The only comprehensive revision of *Albuca* is that of (Baker (1897, 1898), who focused on South African and tropical African taxa. Recent accounts (cf. Müller-Doblies 1994, 1995, 2006; Manning and Goldblatt 2009) have greatly increased the knowledge of groups with nodding-flowers, namely *Albuca subg. Albuca* and *Albuca subg. Falconera* (Salisb.) Baker. However, information on groups with erect-flowers, e.g. *Albuca subg. Mitrotepalum* U. Müll.-Doblies (= *Albuca sect. Branciona* (Salisb.) J.C. Manning & Goldblatt), has remained almost unchanged from the late 19th century, and is the focus of our current research (Martínez-Azorín et al. in press a, b). In addition, no identification keys are available for all currently accepted taxa of *Albuca*, most of which are still poorly known or in need of further studies (cf. Phillips 1926; Dyer 1947). For instance, *Albuca caudata* Jacq., a plant described from the Cape, has long been misinterpreted or even ignored. The study of herbarium specimens as well as natural populations of wild plants has revealed the existence of different biological entities to which the name *Albuca caudata* has been applied. In the present contribution, the name *Albuca caudata* is typified to warrant further usage in the sense it was originally published, and data are also presented to describe a new species to which that name was applied erroneously.

## Materials and methods

Herbarium specimens from the following herbaria were studied: BOL, BNRH, GRA, J, K, KEI, KMG, NBG, NH, NU, PEU, PUC, UFH, WIND (acronyms according to Thiers 2011). Moreover, a detailed morphological study of both taxa was undertaken based on plants from natural populations. Authors of the cited taxa follow IPNI (2011).

## Results and discussion

**Figure 1.**
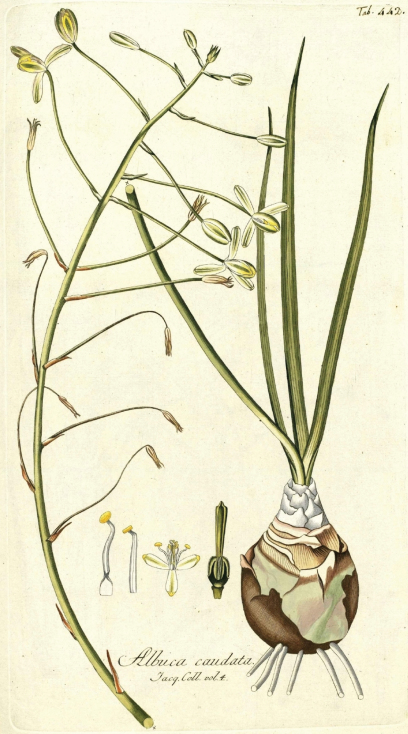
*Albuca caudata* Jacq. from Jacquin, Ic. Pl. Rar. 2(16): 20, t. 442. 1795.

**Figure 2.**
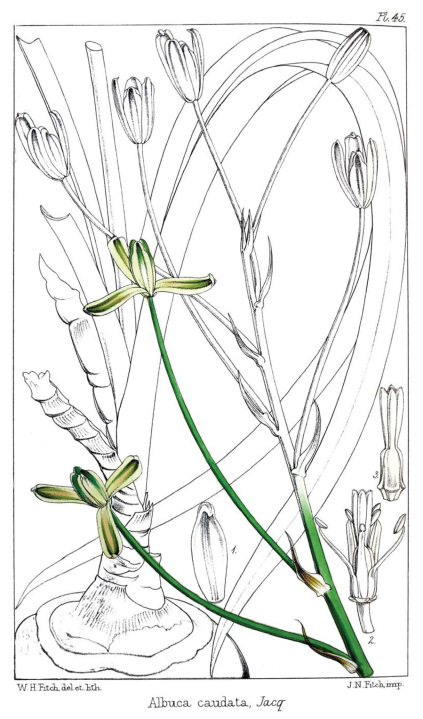
*Albuca bakeri* Mart.-Azorín & M.B. Crespo from Baker, Refug. Bot. (Saunders) vol. 1, t. 45. 1869 (as *Albuca caudata*).

*Albuca caudata* was described by Jacquin (1791) and later illustrated (Jacquin 1795) (Fig. 1) after plants collected in “Promontorio bonae Spei", which “Apud nos in caldariis floret Decembri & Januario". The characters mentioned by Jacquin (1791) are: bulb roundish to ovate; leaves scarce, glabrous, linear-lanceolate, canaliculate, acute, with terete apex; stem weak and inclined; raceme lax, with patent pedicels; tepals white with green bands, the inner tepals with yellowish apices; all six stamens bearing anthers; and style clavate, thick and trigonous. Plants fitting this description are widespread in the Eastern Cape. They are usually easy to recognise by their solitary partially hypogeal bulbs, covered by short, papery, brown to grey scales that reach different heights, and their inclined unilateral racemes with all pedicels erect. However, a wide range in variation of vegetative and reproductive features can be observed, such as morphology of the scales of the bulb neck, leaf length and width, and flower disposition with regard to the inflorescence axis. A number of herbarium specimens labelled *Albuca caudata* differ significantly from Jacquin's description, but match Baker's concept of that taxon (cf. Baker 1869). These plants are characterized by their hypogeal roundish bulbs with fleshy outer tunics, all of them reaching the top of the bulb and ending into a long epigeal neck surrounded by transversally banded cataphylls. The particular characteristics of those collections, which were illustrated by Baker (1869) (Fig. 2), do not fit any of the known species of *Albuca subg. Mitrotepalum*. As differences with the typical *Albuca caudata* are remarkable, segregation at the species rank is here favoured and a new species is described for them. Information on both taxa is provided below.

### Albuca caudata

Jacq., Collectanea 4: 203 (1791).

http://species-id.net/wiki/Albuca_caudata

#### Neotype (here designated).

Jacquin, Ic. Pl. Rar. 2(16): 20, t. 442 (1795), ex Promontorio bonae Spei. Apud nos in caldariis floret Decembri & Januario (Fig. 1).

#### Epitype (here designated).

SOUTH AFRICA. **Eastern Cape:** Alexandria, Addo National Park, 400 feet, 29.X.1954, *S.M. Johnson* 1077 (GRA).

#### Description.

**Figure 3.**
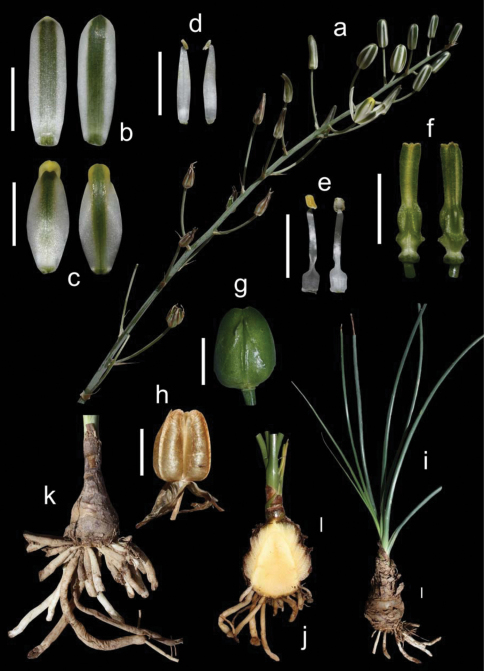
*Albuca caudata* Jacq. Eastern Cape, Redhouse (M. Martínez-Azorín, A.P. Dold & A. Martínez-Soler 45 GRA) **a** Inflorescence **b** Outer tepals **c** Inner tepals **d** Outer stamen **e** Inner stamen **f** Ovary, lateral views **g** Mature capsule **h** Dehiscing capsule **i** Bulb and leaves **j** Bulb in longitudinal section **k** Bulb with tuberose roots. Scales 1 cm.

Evergreen bulbous plants. Bulb mostly solitary and hypogeal, ovoid to oblong, up to 10 × 6 cm, usually with its wide basal plate elongated into a domed axis where the fleshy scales are attached; tunics fleshy, short and usually not reaching the top of the bulb, imbricate, persistent, papery grey or brownish in the upper part, sometimes with transversal prominent dark ridges, giving a brownish multiscaled appearance to the bulb in outline. Roots fleshy, thick and usually tuberose, white, numerous, up to 200 × 4 mm. Leaves 4-10, disposed in an apical rosette, linear-lanceolate, 15-120 × 0.5-2 cm, straight up and curving down when old, infolded, canaliculate, persistent, pale bright green to glaucous, glabrous, usually minutely papillate on nerves and margins, with a terete apex evident in young leaves. Inflorescence inclined, unilateral raceme, 11–40 cm long; peduncle 12–55 cm long; pedicels 3.5–9 cm long at base becoming smaller, up to 0.2–1 cm long near top, patent and being usually all erect; bracts ovate-lanceolate to triangular, long acuminate, 11–25 × 5–9 mm, papery white with brownish distant nerves that converge at the tips, much shorter than pedicels in the lower part of the inflorescence. Flowers erect; tepals white with a green median stripe 2–4 mm wide, sometimes with the tips yellowish; outer tepals oblong, 18–28 × 4–7 mm, apex slightly cucullate; inner tepals ovate, 15–24 × 4–10 mm, with apex strongly cucullate. Stamens all six bearing fertile anthers; outer anthers 1.5–2.5 mm long; inner anthers 3–4 mm long; outer filaments 10–16 × 1.5–2 mm, linear lanceolate to narrowly oblong, not pinched down; inner filaments 10–17 × 1.5–3 mm, linear oblong, wider and pinched in the lower half. Ovary oblong to obovate, up to 6–8 × 2–3.5 mm, stipitate, with prominent paraseptal crests that are divergent in the lower part and form three prominent ridges; style subobpyramidal or clavate, trigonous, up to 7–10 × 2 mm, stigma yellowish green. Capsule ovate, 14–20 × 10–14 mm, trigonous to subsphaerical in section, pale-brown when mature; valves splitting in the upper quarter. Seeds flat, c. 5–6 × 4–5 mm, dark brown to black, flattened and semidiscoidal, biseriate and horizontally stacked in each locule. (Fig. 3)

#### Flowering time.

September to November; capsules dehiscing at the end of November and December.

#### Habitat.

Plants of *Albuca caudata* are often associated with bush-clumps, where the inclined inflorescence is supported by woody plants.

#### Distribution.

**Figure 4.**
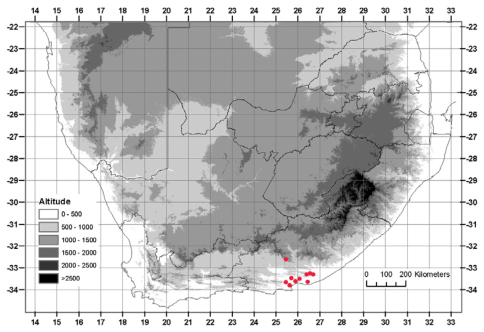
Known distribution of *Albuca caudata* Jacq.

Currently known from Addo in the west to Grahamstown in the east, below 600 m, with an outlying population as far inland as Somerset East, reaching 900 m (Fig. 4).

#### Diagnostic characters.

*Albuca caudata* can be easily identified by its bulb mostly solitary covered by brownish papery scales usually disposed at different heights and bearing long thick tuberose roots, its long and narrow canaliculate or infolded leaves, its inclined raceme, with usually all pedicels patent and erect, giving a unilateral appearance to the inflorescence, and its white erect flowers with a median green stripe (Fig. 3).

#### Etymology.

The specific epithet ‘*caudata*' presumably refers to the rather pointed, tail-like leaves, although Jacquin did not specifically mention it (E.E.A. Gledhill, unpubl. ms. in NBG).

#### Relationships.

The recently described *Albuca batteniana* Hilliard & B.L. Burtt (Hilliard and Burtt 1985) shares some morphological characters with *Albuca caudata*, such as the inclined scape bearing a horizontally arcuate inflorescence with erect pedicels, and the flower morphology. This species, however, differs in the coriaceous recurved much broader and flattened leaves, the longer tepals (30–42 mm long), and the structure of the bulb, being proliferous, epigeal, and composed by scales truncate at the top, disposed into a long domed axis and ending at different heights, without membranous neck (Table 1).

#### Observations.

*Albuca caudata* shows some variability in the colour of the membranous bulb scales, being pale coloured with orange transversal ridges in some inland populations whilst those from the coastal areas are usually brown coloured with darker transversal ridges.

#### Selected specimens studied.

SOUTH AFRICA. Alexandria, 4 miles east of Sandflats, 1000 feet, 17/12/1953, *E.E.A. Archibald* 5431 (GRA); Eastern Cape, along road in Springs Reserve, north of Uitenhage, 39 m, 21.X.2009, *A.B. Low* 16732 (GRA); Addo Elephant National Park, in main Botanical Reserve, 20.X.1996, *K. Johnson* 241 (GRA); Alexandria, Nanaga, opposite Glen Rosa turn-off, 1100 feet, 23.X.1953, *E.E.A. Archibald* 5315 (GRA); Eastern Cape, Albany, Queen´s Road, 10 miles north of Grahamstown, 2000 feet, 05.X.1953, *S. Johnson* 774 (GRA); Eastern Cape, Albany, a few yards from *Archibald* 5636, Pluto´s Vale, 2000 feet, 22.IX.1954, *E.E.A. Archibald* 5636 (GRA); Eastern Cape, Grahamstown, c. 5 miles on Cradock road, 626 m, 11.XII.2009, *M. Martínez-Azorín & A.P. Dold* 85 (GRA); Eastern Cape, Redhouse, thicket west of village, 6 m, 27.XI.2009, *M. Martínez-Azorín, A.P. Dold & A. Martínez-Soler* 45 (GRA).

**Table 1.** Main diagnostic characters among *Albuca caudata*, *Albuca bakeri* and *Albuca batteniana*.

	*Albuca caudata*	*Albuca bakeri*	*Albuca batteniana*
Bulb	Mostly solitary	Mostly solitary	Proliferous
	Ovoid to oblong	Ovoid to spherical	Narrowly oblong
	Outer tunics membranous,brown to grey	Outer tunics fleshy,white to yellow	Outer tunics somewhat coriaceous,green to brown
	Mostly hypogeal	Hypogeal	Mostly epigeal
	Imbricate scales mostly ending at different heights	All scales reaching the top of the bulb	Imbricate scales ending at different heights
	Neck absent or short and thick, covered by brown to grey membranous scales	Neck long and thin, covered by transversally banded sheathing cataphylls	Neck usually absent
Roots	Usually numerous, thick and tuberose	Thin and scarce	Thin or slightly thickened
Leaves	Narrow, infolded and canaliculate	Narrow, infolded and canaliculate	Wide, flattened and usually recurved
Inflorescence	Inclined and secund	Erect and helicoidal	Inclined and secund
Outer tepals	18–28 mm	19–23 mm	30–42 mm
Seeds	5–6 × 4–5 mm	4–5 × 3–4 mm	5–7 × 4–5 mm

### Albuca bakeri

Mart.-Azorín & M.B. Crespo sp. nov.

urn:lsid:ipni.org:names:77112770-1

http://species-id.net/wiki/Albuca_bakeri

#### Holotype.

SOUTH AFRICA. **Eastern Cape:** North of Grahamstown, on Cradock Road turn off to Kwandwe, 592 m, 05.IX.2010, 33°12'39"S, 26°24'07"E, *M. Martínez-Azorín & A. Martínez-Soler* 218 (GRA Holo.; ABH, K, NBG, PRE Iso.).

*Diagnosis*: Species insignis ex *Albuca subg. Mitrotepalum* characteribus floralibus ad *Albucam caudatam* accedit, sed valde differt et facile distinguitur bulbo hypogaeo solitario carnoso tunicis omnibus apicem attingentes in collum angustum supra solum desinentes, e basi cataphyllis albido-membranosis manifeste transversaliter fusco-striatis obtectum qui habitum pulchre zebrinum exhibent, insuper racemo subdeltoideo non secundo floribus spiraliter dispositis.

**Figure 5.**
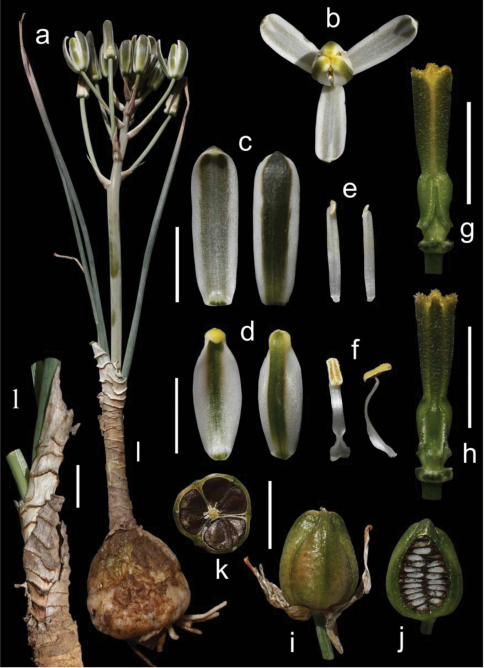
*Albuca bakeri* Mart.-Azorín & M.B. Crespo. North of Grahamstown, turn off to Kwandwe (holotype: *M. Martínez-Azorín & A. Martínez-Soler* 218 GRA) **a** Plant **b** Flower **c** Outer tepals **d** Inner tepals **e** Outer stamen **f** Inner stamen **g–h** Ovary, lateral views **i** Mature capsule **j** Capsule, longitudinal section **k** Capsule, transversal section **l** Bulb neck with membranous banded cataphylls. Scales 1 cm.

*Illustrations*: Baker (1869) in *Refugium Botanicum*, vol. 1, tab. 45 (Fig. 2); Fig. 5.

#### Description.

Evergreen or deciduous bulbous plants. Bulb mostly solitary, occasionally growing in small clumps, hypogeal, ovoid to spherical, 3.2–7 × 2.5–6.5 cm, with soft outer tunics that are pale and fleshy, ending in a long epigeal neck, up to 10 × 2 cm, covered with whitish open and sheathing membranous cataphylls bearing transversal sinuous ridges with their lower side pale to dark brown coloured, giving a zebra banding horizontal pattern; tunics fleshy, whitish, all reaching the top of the bulb, concentrically arranged. Roots fleshy, narrow, white, up to 90 × 2 mm. Leaves 2-6, disposed in an apical rosette, linear-lanceolate to oblong, 9-40 × 0.4-1.3 cm, erect when young and later curving downwards, infolded, canaliculate, persistent or usually deciduous, pale bright green to glaucous, glabrous, usually minutely papillate on nerves and margins, exceptionally with long papillate margins. Inflorescence an erect raceme or subcorymb, 3–15 cm long; peduncle 9–22 cm long; pedicels helicoidally disposed, 3–7.5 cm long, longer at the base, up to 0.2–0.7 cm long near top, erect-patent; bracts ovate-lanceolate to triangular, long acuminate, 9–27 × 4–10 mm, papery white with brownish separated nerves that converge at the tips, much shorter than pedicels at least in the lower part of the inflorescence. Flowers erect; tepals white with a green median stripe 2–3 mm wide, sometimes with the tips yellowish; outer tepals lanceolate-oblong, 19–23 × 5–7 mm, with apex slightly cucullate; inner tepals ovate, 13–17 × 6–7 mm, with apex strongly cucullate. Stamens all six bearing fertile anthers; outer anthers 1.5–3 mm long, inner anthers 4–6 mm long; outer filaments 10–13.5 × 1.5–2 mm, linear lanceolate to narrowly oblong, not pinched down; inner filaments 10.5–14.5 × 2–3.5 mm, linear oblong, wider and pinched in the lower half. Ovary oblong to obovate, up to 6–7 × 2–3.5 mm, stipitate, with prominent paraseptal crests that are divergent in the lower part and form three prominent ridges; style subobpyramidal or clavate, trigonous, up to 7–11 × 3.5–4.5 mm, stigma yellowish green. Capsule ovate, 14–16 × 11–12 mm, trigonous to subsphaerical in section, pale-brown when mature; valves splitting in the upper quarter. Seeds flat, c. 4–5 × 3–4 mm, dark brown to black, flattened and semidiscoidal, biseriate and horizontally stacked in each locule. (Fig. 5)

#### Flowering time.

July to September; capsules dehiscing at the end of September and November.

#### Habitat.

*Albuca bakeri* is found growing singly in dry, stony, open ground at low altitude reaching c. 650 m.

#### Distribution.

**Figure 6.**
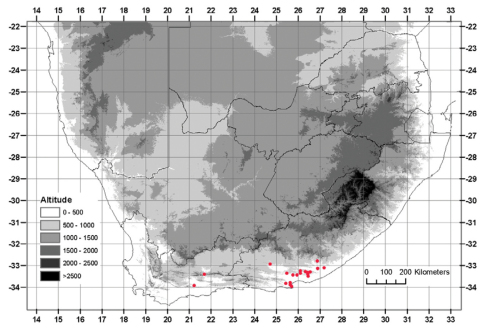
Known distribution of *Albuca bakeri* Mart.-Azorín & M.B. Crespo.

from Jansenville to Alice and the Keiskamma river in the Eastern Cape, with two outlying populations near Calitzdorp in the Western Cape karroo (Fig. 6).

#### Diagnostic characters.

*Albuca bakeri* can be easily identified by its solitary hypogeal fleshy bulb ending in an epigeal neck, covered by whitish transversally banded membranous cataphylls, giving a conspicuous zebra banding pattern (Fig. 5). Moreover, its erect and helicoidal raceme with white and green erect flowers, and the smaller seeds (c. 4–5 × 3–4 mm), separate it from *Albuca caudata*.

#### Etymology.

Name honouring John Gilbert Baker (1834–1920), a leading expert on monocotyledons, who worked at the Royal Botanic Gardens, Kew, and was the keeper of the herbarium K.

#### Relationships.

No other *Albuca* with erect flowers have been described with the characteristic long, thin, zebra banded bulb neck of *Albuca bakeri*. The closest species appears to be *Albuca caudata*, though the structure of the bulb and inflorescence clearly distinguish them (Table 1).

#### Observations.

The peculiar zebra banded cataphylls of *Albuca bakeri* are similar to those found in some other groups of Hyacinthaceae. As pointed out by Müller-Doblies and Müller-Doblies (1981), zebrine cataphylls are present in evolutive distant taxa such as *Rhadamanthus fasciatus* B. Nord., *Tenicroa exuviata* (Jacq.) Speta (Urgineoideae), several species of *Ledebouria* Roth (Scilloideae), *Coilonox zebrinum* (Baker) Speta and some species of *Nicipe* Raf. [= *Ornithogalum* sect. *Vaginaspasia* U. Müll.-Doblies & D. Müll.-Doblies] (Ornithogaloideae). Moreover, *Stellarioides arida* (Oberm.) Speta and *Battandiera stapffii* (Schinz) Mart.-Azorín, M.B.Crespo & Juan show the neck of the bulb covered with membranose transversally banded cathaphylls, indicating that the zebrine cataphylls could have evolved independently in at least five lineages of the Ornithogaloideae (e.g. *Albuca*, *Battandiera*, *Coilonox*, *Nicipe* and *Stellarioides*), possibly as a result of convergent evolution in dry climates of southern Africa.

Some morphological variation has been found within *Albuca bakeri*. Some individuals from Janseville and Port Elizabeth have a slightly setose bulb neck with the characteristic transversally banded membranous cataphylls of this species. Other specimens from Grahamstown and Port Elizabeth showed somewhat proliferous bulbs, resulting in a small clump of plants growing together, and with shorter scales not so markedly banded.

When Baker (1897) described and illustrated his concept of *Albuca caudata* (Fig. 2), he mentioned: “Bulb two to three inches thick, round or oblong, crowned as in the preceding [*Albuca fastigiata* Dryand.] with brown fibres. Leaves about a foot long, four lines broad, more rigid than in the preceding, clasping the stem at the base and more or less concave on the face upwards, and keeled on the back". This description is vague and inaccurate, since the illustration he presented did not show “fibres" at all, and no specific comments on the transversal banding of the upper scales of the bulb were made. However, when Baker reconsidered *Albuca caudata* in later works, his previous concept was changed to “Bulbus globosus 2–3 poll. crassus viridis apice squamosus" (Baker 1872), or “bulb globose, 2–3 in. diam.; tunics not splitting into fibres at the top" (Baker 1897), or “Bulb globose, 2–3 in. diam." (Baker 1898).

#### Materials studied.

SOUTH AFRICA. Eastern Cape, Alexandria, 1½ miles east of Paterson, 1000 feet, 24.VIII.1953, *E.E.A. Archibald* 5972 (GRA); Eastern Cape, Alexandria, south-west end of Zuurkop, Addo National Park, 1000 feet, 23.IX.1953, *S.M. Johnson* 751 (GRA); Eastern Cape, Albany, 5 miles north of Alicedale, on Riebeck East road, 1500 feet, 21.IX.1954, *E.E.A. Archibald* 5638 (GRA); Victoria East: Alice, dry stony places on Sandilis Kop on north east side, 08.IX.1934, *M.H. Giffen* 614 (GRA); Victoria East: Alice, Sandilis Kop western side among grass, 13.IX.1935, *M.H. Giffen* 618 (GRA); Hillside, Gowie´s Kloof, Grahamstown, IX.1947, *Hill* s.n. (GRA); Grahamstown, West Hill, Pine plantation, VIII.1956, *V. van Niekerk s.n.* (GRA); Cradock road, Grahamstown, 01.IX.1945, *E. Barrat* 28 (GRA); In graminosis prope Grahamstown, *M. Daly & M. Sole* 316 (BOL); In graminosis prope Grahamstown, 2000 feet, VIII.1893, *Schonland s.n.* (NBG); Grahamstown (3326 BC): Ecca Reserve, south near old Queens Road/Quarry, 20.VIII.1992, *T. Dold* 153 (GRA); Leander Beacon, VIII.1943, *L. Miles s.n.* (GRA); Port Elizabeth, Summerstrand, grassy roadside, IX-X.1990, *H.J. Vanderplank s.n.* (GRA); Port Elizabeth (3325CD): 3 km south of Uitenhage towards van Stadens, 01.IV.1978, *P.L. Perry* 601 (NBG); Port Elizabeth (3325CB): Kirkwood District, farm Brakleegte, 300 m, 28.VIII.1985, *M.T. Hoffman* 1064, 1065 (NBG); *ibidem*, 14.IX.1985, *M.T. Hoffman* 1002 (NBG); Graaff-Reinet (3224DC): District Janseville, just south of Janseville (+/- 1 km) in municipal-owned land, 11.VIII.1985, *M.T. Hoffman* 1063 (NBG); Ladismith (3321BC): Calitzdorp dam, 22.II.1981, *P.L. Perry* 1521 (NBG); Ladismith (3321CC): Sopieshoogte, north entrance to Garcia´s Pass, Riversdale, 1600 feet, 15.IX.1981, *Albuca Fellingham* 149 (NBG); Eastern Cape, Grahamstown, hills above Botanic Garden, 591 m, 14.XI.2009, 33°19'04"S, 26°31'15"E, *M. Martínez-Azorín & A. Martínez-Soler* 12 (GRA); Eastern Cape, Grahamstown, Burnkraal, 649 m, 24.XI.2009, 33°16'40"S, 26°29'41"E, *M. Martínez-Azorín & A.P. Dold* 34 (GRA); Eastern Cape, Redhouse, thicket west of village, 6 m, 27.XI.2009, 33°50'01"S, 25°33'56"E, *M. Martínez-Azorín, A.P. Dold & A. Martínez-Soler* 44 (GRA); Eastern Cape, north of Grahamstown, Table Hill farm, 587 m, 11.XII.2009, 33°15'21"S, 26°27'17"E, *M. Martínez-Azorín & A.P. Dold* 83 (GRA); Eastern Cape, north of Grahamstown, on Cradock road turn off to Kwandwe, 594 m, 31.I.2010, 33°12'38"S, 26°24'07"E, *M. Martínez-Azorín, M.B. Crespo & A. Martínez-Soler* 118 (GRA); Eastern Cape, Quamnyana, between Breakfast Vlei and Commitees Drift, 411 m, 14.VIII.2010, 33°06'56"S, 22°55'57"E, *C. Peter* (GRA); *ibidem*, 27.VIII.2010, *M. Martínez-Azorín & A.P. Dold* 207 (GRA); Eastern Cape, Port Elizabeth, Settler´s Park, 28 m, 03.IX.2010, 33°58'21"S, 25°36'09"E, *M. Martínez-Azorín & A.P. Dold* 210 (GRA); Eastern Cape, Grahamstown, Sunny side, Hillsview street, 570 m, 07.IX.2010, 33°19'08"S, 26°32'00"E, *M. Martínez-Azorín & A. Martínez-Soler* 221 (GRA); Eastern Cape, Alicedale, railway cross to Burchell Game Reserve, 288 m, 14.IX.2010, 33°18'51"S, 26°06'01"E, *M. Martínez-Azorín & A. Martínez-Soler* 226 (GRA); Eastern Cape, Alice, Fort Hare University, Sandili's Kop, 582 m, 17.IX.2010, 32°47'02"S, 26°51'38"E, *M. Martínez-Azorín & A. Martínez-Soler* 235 (GRA); Eastern Cape, Keiskamma River, Linedrift, 141 m, 12.XI.2010, 33°04'29"S, 27°13'02"E, *M. Martínez-Azorín, A.P. Dold & A. Martínez-Soler* 525 (GRA).

## Supplementary Material

XML Treatment for Albuca caudata

XML Treatment for Albuca bakeri
